# Avoiding Chest Wall Morbidity in Outpatient Microvascular Free-Flap Breast Reconstruction

**DOI:** 10.3390/jcm14020602

**Published:** 2025-01-18

**Authors:** Carlos A. Martinez, Sean G. Boutros

**Affiliations:** My Houston Surgeons, 9230 Katy Freeway, Suite 600, Houston, TX 77055, USA; drcmart@gmail.com

**Keywords:** breast reconstruction, DIEP flap, rib sparing, internal mammary, perforator, microsurgery

## Abstract

**Background.** Removal of the rib and adjacent cartilage is a common step for exposure of the recipient chest vessels in free-flap breast reconstructions. However, this adds both short- and long-term morbidity to the procedure. We describe our experience in avoiding rib removal in microvascular breast reconstruction. **Patients and Methods.** We retrospectively reviewed recipient vessel preparation in free-flap breast reconstructions performed by a single surgeon (SGB). **Results.** A total of 556 consecutive patients, totaling 1106 flaps over 5 years, were assessed. Recipient vessels included IMA in 1068 flaps and internal mammary perforator in 38 DIEP flaps. Nine patients underwent bilateral DIEP flap breast reconstruction with a cross-chest anastomosis, where the IMA was the recipient. Also, the IMA was used in 171 patients who underwent breast reconstruction with stacked flaps. No instances of complete rib resection were reported. However, in two cases of delayed DIEP flap reconstruction without a history of radiation, resection of 20% of the rib was required for safe vessel preparation. No intraoperative complications were observed, and three flaps from different patients were lost (one PAP and two DIEPs). **Conclusions.** Microsurgery in free-flap breast reconstructions has greatly evolved in the past two decades. Exposure of the IMA recipient vessels typically involves the removal of a portion of the intercostal cartilage and the rib, allowing comfortable and safe management of the vasculature during dissection and anastomosis. Nonetheless, excessive removal often leads to short-term increased pain and long-term cosmetic and functional complications, such as a noticeable depression of the chest wall especially noted in thin patients with small flaps. Our approach can be safely employed to preserve the anatomy and decrease pain, allowing for outpatient performance of these procedures.

## 1. Introduction

Perforator-based free flaps have become the gold standard in microvascular breast reconstruction, reducing the morbidity of the donor sites while providing outstanding esthetic results. However, free flaps like the deep inferior epigastric perforator [1], the profunda artery [2], or the gluteal artery perforator [3] require exposure and preparation of the recipient chest vessels before the anastomosis. While the variety and availability of donor tissues are advantageous to patients and surgeons, the selection of the recipient vessels is rather limited, often between the internal mammary or the thoracodorsal vessels [4]. The internal mammary vessels (IMVs) offer many advantages, such as straightforward access, central location, and uniform quality, making them the most favored choice [5]. For most surgeons, this approach typically requires the removal of a portion of the rib, leading to both short- and long-term complications, chief among them being chronic pain and anatomical defects of the chest wall. While preservation or removal of the rib and cartilage plays no major role in the survival of the flap, a foremost concern to the surgeon, it does account for the overall success of the reconstruction in terms of cosmesis and postoperative satisfaction. Only a few reports have addressed the conservation of the rib and cartilage to preserve the chest anatomy, albeit briefly, and have overlooked the myriad of situations and potential issues that can be encountered [5,6,7,8,9,10]. We hypothesize that preservation of the rib and costal cartilage, when performed by experienced surgeons, is not associated with an increase in the risk of damage to the recipient vessels during preparation and dissection or a significant increase in operating times. Moreover, we describe our experience and thought process when approaching the recipient vessels and the technical particularities.

## 2. Patients and Methods

### 2.1. Patients and Data Collection

We performed a retrospective analysis of patients undergoing immediate and delayed breast reconstruction with perforator flaps by the senior author (S.G.B.) over 5 years. A minimum of 12 months of postoperative follow-up was required for inclusion. A modified recovery protocol, previously reported by the authors in [11,12], was applied and included a rib- and cartilage-sparing approach to dissect and prepare the recipient chest vessels, accompanied by the minimally invasive harvest of the flap, intraoperative pain control, and an anticoagulation regimen. This protocol allowed our patients to be discharged within 23 h of surgery, effectively performing microvascular breast reconstructions in an outpatient setting.

Variables assessed include age, indication for mastectomies, comorbidities, body mass index, and smoking history. Likewise, we reviewed information relating to the location and configuration of the harvested tissue, laterality, and chosen recipient chest vessels. Complications of the flaps and donor sites were also reported.

### 2.2. Internal Mammary Vessels Description

The internal mammary—or internal thoracic—vessels are the preferred recipient in breast reconstructions with perforator flaps due to their location, easy access, caliber, and usual quality. The internal mammary artery (IMA) is a paired vessel originating from the proximal subclavian artery, with a cranial trajectory from the clavicle to the level of the sixth rib and traveling underneath the costal cartilages. The paired vessels are normally located 1.5 cm parallel to the sternal border; the distance increases as it descends, ranging from 0.2 to 2.5 cm [13]. The IMA has its widest caliber, around 2.5 mm, between the third and fifth intercostal spaces, accompanied by two medial venae comitantes that typically form a single vein in 70% of patients between the second and third intercostal spaces [14,15]. The IMA provides several collateral branches that supply the superior aspect of the abdominal wall and the anterior aspect of the thoracic cage. Likewise, several perforators travel through the intercostal spaces, and at the level of the second intercostal space, larger perforators can be found with mean diameters of 1.5 mm which can be used as recipient vessels. The IMA eventually bifurcates into the musculophrenic and the superior epigastric arteries below the sixth intercostal space.

### 2.3. Identification and Exposure of the Intercostal Space

While the selection of the intercostal space relies on the surgeon’s preference, the senior author often favors the fourth intercostal space as the lower intercostal spaces are more easily accessed via inframammary incision and the lower location decreases the risk of deformity at the pectoralis division site. Although the fifth intercostal space may be a goal choice in many cases, it is technically challenging as the space can be exceedingly narrow. We use the third intercostal space as needed in emergencies. Nonetheless, careful selection should be considered to prevent potential inconveniences, as seen in secondary breast reconstructions or post-radiation patients which can present with a distorted anatomy. Preoperative examination and marking are personal preference, although a good landmark is found at the angle of Louis, directly adjacent to the second rib cartilage.

### 2.4. Creation of the Intercostal Window

**Figure 1:**
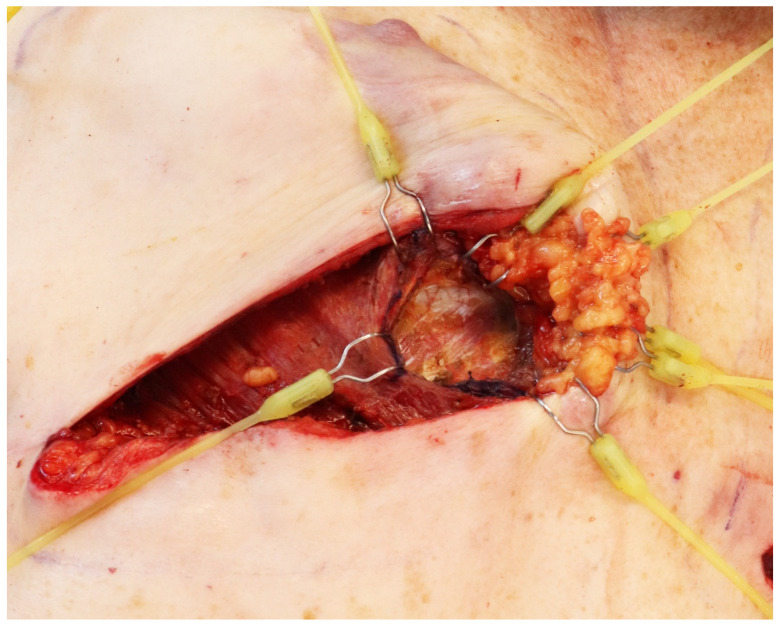
Retraction of soft tissues is facilitated using double-hooked elastic stays, easily moved and repositioned to accommodate the surgical field.

**Figure 2:**
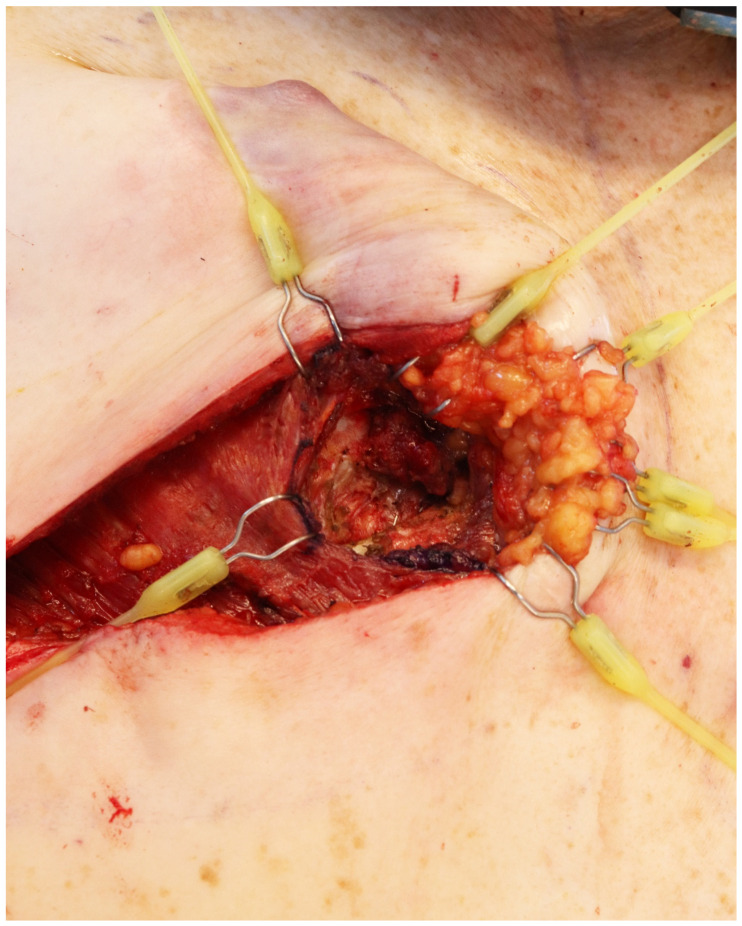
Exposure and dissection of the intercostal space.

An L-shaped full-thickness division of the pectoralis major is performed with the monopolar cautery. We do not recommend overextending the division of the major pectoralis laterally and emphasis should be set on medial exposure. Retraction of the skin flap and both pectoralis muscles can be facilitated using double-hooked elastic stays as they are easily moved and repositioned to accommodate the surgical field (Figure 1); multi-lock retractors and sutures could also be used for retraction, though high tension should be avoided to lessen damage to the skin. As the intercostal space is exposed, a two-to-three-centimeter *intercostal muscle window* is retracted or removed with bipolar electrocautery, revealing the internal mammary artery. Hemostasis is maintained with diathermy, raising the flap and retracting it laterally with an elastic stay (Figure 2). Small branches can be found during the division of the intercostal muscles, which can be controlled with electrocautery or bipolar electrocautery.

### 2.5. Exposure and Dissection of the IMV

**Figure 3:**
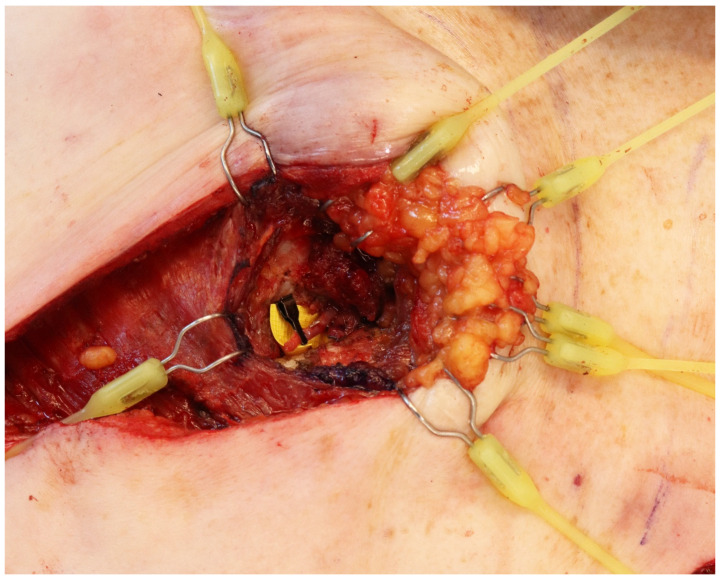
Microvascular clamps are used to secure the recipient vessels.

The IMV will be visualized once the intercostal muscles are divided. These vessels are marked for reference and, under the microscope or loupe magnification, bluntly dissected from their connective tissue while side branches are controlled with micro-clips, devoting special attention to avoiding over-manipulation of the vessels and preserving the intercostal innervation. Once they have been released and bisected, microvascular clamps are used to secure the vessels followed by preparation of the flap and anastomosis in the usual fashion. In our experience, the IMV can be accessed and safely dissected in an intercostal window as narrow as 5 mm (Figure 3).

Regarding the use of internal mammary perforators as recipient vessels, adequate perforating arteries are rare in our experience. We do not recommend this course in immediate reconstructions when the vessels have not been previously divided by the breast surgeon for fear of mastectomy flap ischemia. Furthermore, we have not found a reduction in overall operative times, as their preparation required additional attention, but can be useful in cosmetic augmentation with DIEP flaps as their use will limit the inframammary incision.

Finally, due to the lateral flap placement and potential scarring of the axilla, the thoracodorsal artery (terminal branch of the subscapular artery) as a recipient vessel has become an infrequent choice in immediate reconstructions. Moreover, the decline in axillary node dissections in favor of sentinel lymph node biopsies has positioned the thoracodorsal system as an alternative only used in exceedingly rare circumstances.

### 2.6. Cross-Chest Anastomosis

Another pathway that can be taken to further minimize chest wall morbidity in bilateral reconstructions can be achieved by tunneling the pedicle of one of the flaps over the sternum and anastomosing it to the contralateral flap. The primary flap is set in the usual manner with dissection and preparation of the recipient vessel without removal of the rib, whereas in the contralateral—or secondary—side, only the chest pocket is created without division of the pectoralis or dissection of the underlying anatomy. As mentioned, a narrow tunnel is then created across the chest where the pedicle of the secondary flap is brought and anastomosed to the primary flap in a sequential anastomosis. The authors reported a small series of patients who underwent this procedure, performed for symmetry purposes in cases of unilateral breast cancer and bilateral failed prostheses [16]. While its use in patients with bilateral mastectomies is yet to be determined, we believe that its potential to limit postoperative discomfort and potential chest depression is worth considering. Conversely, in cosmetic DIEP flaps where the incision is limited, especially in primary augmentations that do not require a lift, the cross-chest approach has proven invaluable in our practice.

### 2.7. The Radiated Chest and Final Considerations

The impact of radiation on the soft tissues of the chest is widely known, with injuries typically manifesting in two phases: acute and chronic. The acute phase is described as an inflammatory reaction that includes cutaneous erythema and edema, often accompanied by desquamation and ulceration. The chronic phase is diverse in terms of presentation and severity, but most patients present with induration or retraction of the skin, atrophy and fibrosis of tissues, and radiation-induced vasculopathy. The latter is characterized by potential narrowing and inelasticity of the recipient vessels, relative to radiation dose, which can potentially lead to intra- and postoperative vascular complications. If the surgeon encounters friable internal mammary vessels with damage to the intima during inspection or even at the time of the anastomosis, the safest choice is to shift to a non-radiated recipient vessel (e.g., thoracodorsal) to avoid flap complications. Interestingly, Baumann et al. [17] found higher complication rates in left-sided reconstructions following radiotherapy, perhaps due to the inherent small caliber of the vessels when compared to their right-sided counterparts. While this has not been our experience, this could complicate the technical aspect of the dissection and preparation if encountered.

## 3. Results

We assessed 556 consecutive patients undergoing breast reconstruction with autologous tissue over 5 years, totaling 1106 flaps. Flaps included DIEP, PAP, and gluteal artery perforator (GAP), as well as stacked configurations in cases of inadequate tissue availability. Table 1 summarizes demographics, flap characteristics, and operative courses. Recipient vessels included IMA in 1068 flaps and internal mammary perforator in 38 DIEP flaps. Nine patients underwent bilateral DIEP flap breast reconstruction with a cross-chest anastomosis, where the IMA was the recipient. Also, the IMA was used in 171 patients who underwent breast reconstruction with stacked flaps. No instances of complete rib resection were reported, although in two cases of delayed DIEP flap reconstruction without a history of radiation, resection of 20% of the rib was required to allow for a safe vessel preparation. The surgical times required to prepare the recipient vessels were approximately 15 min, although precise times were not recorded. All patients were discharged within 23 h of admission following our outpatient protocol for breast reconstruction. One bilateral DIEP reconstruction was taken back three hours after surgery due to a sudden loss of signal on Doppler ultrasound and congestion concerns, although no compromise of the anastomosis was found during exploration and the patient followed an uneventful postoperative course. Two patients were readmitted: one with severe dyspnea and a history of pulmonary embolism which was resolved uneventfully, and another with acute enlargement of the reconstructed breast (internal mammary artery perforator) on postoperative day four due to an expanding hematoma, safely evacuated and without further complications. Three flaps from different patients were lost, two DIEPs and one PAP (all IMA), secondary to venous congestion. These patients were discharged with strong Doppler signals and no signs of vascular compromise; the first DIEP flap was initially assessed two days postoperatively, with evidence of vascular congestion and loss of Doppler signal, unsuccessfully salvaged. The second DIEP flap, which underwent IMA anastomosis, was assessed on postoperative day 5 without signs of vascular injury and strong Doppler signals, but during a reinspection on postoperative day 9 it presented clear signs of edema with loss of Doppler signal. Of note, this patient had a history of miscarriages and a coagulation disorder was suspected. The lost PAP flap had a sequential anastomosis, and signs of venous congestion were observed the day after surgery, where salvage attempts were unsuccessful. Donor-site complications included infection (9) and dehiscence of the abdominal wound (28) in varying degrees, which were promptly resolved, while 27 patients required minor debridement of necrotic mastectomy skin flaps. Lastly, one DIEP patient required drainage of a flap abscess without further complications.

## 4. Discussion

Currently, perforator-based flaps like the DIEP or SIEA are the preferred choice for breast reconstruction with autologous tissue. The advantages and drawbacks of these procedures, such as muscle preservation or challenging learning curves, have been discussed at length elsewhere. Likewise, the internal mammary system has become the optimal vascular recipient in both immediate and delayed breast reconstructions with free flaps, and it is almost exclusively used by the senior surgeon in breast reconstructions and autologous cosmetic augmentations. Its central location proves a major advantage over the thoracodorsal vessels, not only by facilitating the recreation of a full central breast pocket but also by allowing for shorter flap pedicles while avoiding a far-from-cosmetic dissection of the axilla.

Regardless, the microvascular nature of free flaps requires the use of recipient vessels, and even though the dissection and preparation of the internal mammary vessels can be considered safe and straightforward, many surgeons traditionally expose and prepare the vessels after resecting a portion of the costal cartilage to facilitate the anastomosis. This is frequently associated with chronic pain and depression of the chest wall, and uncommon instances of intercostal neuralgia or pneumothorax [18,19,20,21]. It should be noted that in a handful of cases, the caliber and position of the internal mammary perforators allow for a direct anastomosis to the flap, which avoids the potential complications aforementioned, and these vessels should be carefully examined at the time of the chest dissection. Nonetheless, we believe that the IMV provides a more reliable anastomosis when compared to its perforators, simply due to an increased flow and larger calibers.

Enhanced recovery protocols have become a cornerstone of the evolving state of microsurgical breast surgery, improving healing and hospitalization times without sacrificing results or increasing overall costs. A core feature of these protocols is the preservation of the anatomy and function, muscle-sparing flaps being a prime example. The idea of saving the costochondral rib to minimize postoperative pain and chest wall deformities was initially described by Levine et al. in 2006 as the “opened book” technique [22], followed by Parrett in a comparative outcome review [6], and Sacks in a series of 100 reconstructions with DIEP and muscle-sparing TRAM flaps [7]. We share the sentiment of the authors regarding this approach in terms of its ample reliability and reproducibility, without increased surgical times or complication rates.

These benefits are further appreciated in the outpatient setting. Two of the most important factors that contribute to extended hospitalizations following microvascular breast reconstructions are continuous supervision of the flap and postoperative pain management. Monitoring the flap is paramount, and we have addressed our opinions concerning this in an outpatient setting in two previous reports [11,12]. Conversely, the postoperative pain typically associated with these procedures is something we should aim to ameliorate. In our opinion, the pain experienced by patients following breast reconstruction with abdominal flaps should be comparable to, if not less than, most abdominoplasties considering the marked pain associated with the abdominal muscle plication, and can be further reduced when the pedicle is dissected through microfascial incisions. Effectively, the resulting pain from the small division of the pectoralis and intercostal muscles can be easily controlled with local anesthetics without hindering early ambulation, thus allowing early recovery times and discharge within 23 h.

It should not be understated that techniques like this come with a steep learning curve. The main limiting factors are the decreased vessel lengths and confined space left to perform the microvascular anastomosis. We believe that experienced surgeons can be comfortable enough handling intercostal spaces of less than one centimeter. Less experienced surgeons can opt for a higher intercostal space with more room or increase the dissection area by removing a small portion of the rib to achieve a safe dissection.

Two limitations encountered during the design of this project were the lack of a control group and recall bias. While the reasoning for this approach is to improve our patients’ cosmetic appearance and postoperative pain management, a control group would help in identifying potential risk indicators, such as BMI and precise surgical times during the dissection and preparation of the recipient vessels, and accurately comparing our outcomes. Since having a control group for the study entailed exposing our patients to the aforementioned potential complications, we chose not to follow this route.

## 5. Conclusions

Excessive removal of the intercostal cartilage or rib can lead to short-term increased pain and long-term cosmetic and functional complications, such as a noticeable depression of the chest wall especially noted in thin patients with small flaps. Our approach can be consistently performed and safely employed to preserve the anatomy and decrease the odds of postoperative pain.

## Figures and Tables

**Table 1:** Patient demographics, flap characteristics and postoperative outcomes.

**Parameter**	**Value**
Patients	556
Age	46.3 ± 8.3 (range 28–77)
Body mass index (BMI)	24.8 ± 3.9 (range 18–41)
Previous abdominal surgery	209 (37%)
Mastectomy due to cancer	501 (90%)
Mastectomy due to benign disease or prophylaxis	55 (10%)
Pre-reconstruction adjuvant therapy	
Radiation	207 (37%)
Chemotherapy	124 (22%)
Radiation + chemotherapy	34 (6%)
Timing of reconstruction	
Immediate	305 (54%)
Delayed	251 (46%)
Flap design	
Bilateral DIEP	334 (60%)
Unilateral DIEP	87 (15.6%)
Bilateral DIEP + PAP	40 (7.1%)
Unilateral DIEP + DIEP	28 (5%)
Bilateral PAP	18 (3.2%)
Unilateral PAP + PAP	41 (7.3%)
Bilateral GAP	2 (<1%)
Unilateral GAP	4 (<1%)
Bilateral DIEP + GAP	2 (<1%)
Complications	
Flap loss	3 *
Hematoma	3
Venous/arterial thrombosis	6
Donor-site complications	38 ^†^
Skin flap necrosis (significant)	27
Other	1
Follow-up in weeks	23 (range 10–30)

* Single flap in 3 different patients; ^†^ including infection, and dehiscence requiring minor debridement.

## Data Availability

The data presented in this study are available on request from the corresponding author. Patient identifiers.
